# Dental approaches in children with congenital heart disease treated under general anesthesia for oral rehabilitation

**DOI:** 10.4317/medoral.26304

**Published:** 2024-05-25

**Authors:** Kevser Kolçakoğlu, Damla İzel Korkut, Gül Yücel, Esra Kızılcı

**Affiliations:** 1 Lecturer, DDS, PhD, Department of Pediatric Dentistry, Erciyes University Faculty of Dentistry, Kayseri, Turkey; 2 Research Assistant, DDS, Erciyes University, Faculty of Dentistry, Department of Pediatric Dentistry, Kayseri, Turkey; 3 Assistant Professor, MD, Pediatrician, Department of Pediatric Dentistry, Erciyes University Faculty of Dentistry, Kayseri, Turkey; 4 Associated Professor, DDS, PhD, Department of Pediatric Dentistry, Erciyes University Faculty of Dentistry, Kayseri, Turkey

## Abstract

**Background:**

Children with congenital heart disease (CHD) are at high risk of contracting oral diseases. The aim of this study is to investigate dental procedures to prevent the risk of infective endocarditis in children with CHD.

**Material and Methods:**

146 patients aged 2-14 years, in need of prophylaxis before cardiovascular surgery and who had filled out anamnesis records, were considered. Dental caries in all the children with CHD was reported as the number of decayed, missing and filled teeth (DMFT).

**Results:**

There was a significant strong positive relationship between the pre-oral rehabilitation DMF-T/dmf-t scores and the number of caries patients (r=0.95, *p*=0.01). There was no significant correlation between the pre-oral rehabilitation DMF-T/dmf-t scores and both tooth loss (r=0.14, *p*=0.09) and the number of restorations (r=0.11, *p*=0.17). In addition, there was no significant correlation between the post-oral rehabilitation DMF-T/dmf-t scores and the prevalence of dental caries. A positive and moderately strong correlation was found between the post-oral rehabilitation DMF-T/dmf-t scores and the number of missing teeth (r=0.56, *p*=0.01), while there was a positive and strong relationship between the post-treatment DMF-T/dmf-t scores and the number of fillings (r=0.62, *p*=0.01).

**Conclusions:**

Extraction should be considered when providing oral rehabilitation, rather than endodontic and deep restorative treatments.

** Key words:**Heart defects, congenital, dental caries, endocarditis.

## Introduction

Congenital heart disease (CHD) is defined as an abnormality in the structure of the major vessels or heart and occurs in 8 out of 1000 live births worldwide (1). Shunting defects in CHD include atrial septal defects (ASDs), ventricular septal defects (VSDs), patent ductus arteriosus (PDA), and atrioventricular septal defects. Stenotic defects in CHD include aortic stenosis, pulmonary stenosis, and coarctation of the aorta. Complex CHD defects include transposition of the great arteries, tetralogy of Fallot (TOF), and hypoplastic left heart syndrome. These account for 90% of heart defects (2). Some drugs that are taken for this disease, especially those containing digoxin, are sucrose-based and associated with caries. In addition, most heart drugs are frequently taken with sweetened food for motivational purposes (3,4).

Due to ameloblasts being very sensitive to alterations in metabolic conditions, CHD could cause developmental enamel defects (5). Furthermore, it has been reported that primary dental enamel hypoplasia is more common in children with CHD than in healthy controls (6). Research has shown that children with CHD have poor oral hygiene. In addition, these children have a much higher prevalence of caries than do healthy children (7) and are more likely to contract oral diseases because of these problems (8).

In children with CHD, odontogenic infections caused by caries or periodontal diseases such as periodontitis and gingivitis can cause infective endocarditis, a life-threatening medical condition (9,10). Specifically, one-third of these children are at higher systemic risk due to episodes of increased bacteremia (11). The oral health status of children with CHD could lead to other systemic complications (12). Therefore, untreated carious teeth should be avoided in children with CHD due to the risk of endocarditis caused by dental sepsis (13). Preventive measures for infective endocarditis should be taken as early as possible in these children (14,15). Consequently, the European Society of Cardiologists and the American Heart Association guidelines precisely described antibiotic prophylaxis to prevent the risk of endocarditis during dental procedures (10,13). Moreover, finding ways to prevent oral health problems has been emphasised in children with CHD (10,16,17). This highlights the importance of good oral health in these children (13). The aim of this study is to investigate dental procedures to prevent the risk of infective endocarditis (IE) in children with CHD.

## Material and Methods

- Study design and participants

Patients aged 2-14 years, in need of prophylaxis before cardiovascular surgery and who had filled out anamnesis records, were referred to Author’s University Faculty of Dentistry, Department of Pedodontics. There they were evaluated for their oral status and need of treatment. This study was performed on 146 patients who came to this department for evaluation from January 2017 to December 2022. The specialist paediatrician of these patients performed systemic and physical examinations, and the patients’ records were included in the study. Additionally, written informed consent was obtained from the parents of the participants. The study was conducted in collaboration with the Department of Pediatrics and Department of Paediatric Dentistry, Authors’ University. The authors of this study are specialists at the Paediatric Dentistry of Authors’ University. According to World Health Organization standards, dental caries from all the children with CHD was reported as the number of decayed, missing and filled teeth (DMF-T) (18).

- Statistical analysis

Descriptive statistics are presented with frequency, percentage, average and standard deviation values. A paired t-test was used to investigate the difference in DMFT levels according to the treatment time was used. A Mann-Whitney U test was applied due to the low number of disease groups in the examination, according to the type of heart disease. Pearson’s test of correlation was applied to examine the relationships between age and the scores of the DMFT levels. In this study, *p-value*s less than 0.05 were considered statistically significant. Analyses were made using the SPSS 22.0 (SPSS Inc., Chicago, Illinois) software.

## Results

It was determined that 56.2% of the patients were boys and 43.8% were girls. In addition, it was determined that 35.6% of the patients had heart and comorbidity disease, 93.2% had heart disease, and 26% used heart drugs. The mean age of the children was 6.86±3.11. Pre-op oral rehabilitation DMF-T/dmf-t scores consist of 1557 caries, 26 missing, and 24 filling in 146 patients. Post-op oral rehabilitation DMF-T/dmf-t scores consist of 0 caries, 525 missing, and 1082 filling in 146 patients. 325 primary and 154 permanent teeth are extracted. 722 primary and 360 permanent teeth are filled (Table 1).

Among comorbidity diseases, Down Syndrome, epilepsy, Ehlers-Danlos and Hypothyroidism, type 1 diabetes mellitus, asthma, attention-deficit/hyperactivity disorder, anaemia, and autism contribute 20.5%, 8.9%, 4.1%, 3.4%, 2.1%, 1.4%,0.7% and 0.7%, respectively. (Table 1).

Table 1 shows that 17.1% of the patients used Enalapril with Acetyl salicylic acid; 10.3% used Depot penicillin; 2.7% used Propranolol HCI and Furosemide; 1.4% used Metoprolol, Risperidone and Digoxin; and 0.7% used Bosentan, Clopidogrel, Atorvastatin, Enoxaparin, Sotalol and Losartan.

Of all the patients, 30.8%, 38.4%, 31.6%, 8.9%, 7.5%, 5.5%, 2.7% and 1.3% had ASDs; VSDs; heart valve anomalies; Williams-Beuren syndrome with heart anomalies; bicuspid aortic valves; myocardial hypertrophy; mitral regurgitation; and aortic valve replacements, acute rheumatıc fevers (previously), arrhythmias or pulmonary venous return anomalies, as shown in Table 1.

In this study, pre-oral rehabilitation DMF-T/dmf-t scores were 10.94±4.14, and post-oral rehabilitation DMF-T/dmf-t scores were 52.68 ± 13.49. It revealed that the patients' pre-oral rehabilitation and post-oral rehabilitation DMF-T/dmf-t scores were statistically significantly correlated. According to the results, the DMF-T/dmf-t scores of the post-treatment increased significantly compared to those of the pre-treatment (*p*=0.01, *p*<0.05).

According to gender, there was no significant difference between the pre- and post-oral rehabilitation DMF-T/dmf-t scores (*p*>0.05, Table 2). According to comorbidity disease, there was no significant difference between the pre- and post-oral rehabilitation DMF-T/dmf-t scores (*p*>0.05). There was also no significant difference in the DMF-T/dmf-t scores between patients with and without comorbidity disease (*p*>0.05). Furthermore, there were significant differences in the pre-treatment DMF-T/dmf-t scores among patients who did not use drugs (*p*=0.01, *p*<0.05). There was no significant difference in the post-oral rehabilitation DMF-T/dmf-t scores among patients who did not use drugs (*p*=0.39, *p*>0.05). Moreover, there were significant differences between the pre-treatment DMF-T/dmf-t scores between patients with and without cardiac surgery (*p*=0.02, *p*<0.05). In addition, there were significant differences in the post-oral rehabilitation DMF-T/dmf-t scores between patients with and without cardiac surgery (*p*=0.21, *p*>0.05).

There were significant differences between the pre- and post-oral rehabilitation DMF-T/dmf-t scores of patients with ASDs, VSDs, mitral regurgitation, bicuspid aortic valves, aortic valve replacements and supravalvular aortic stenosis (with Williams syndrome, myocardial hypertrophy (*p*<0.05, Table 3). There were no significant differences between the pre- and post-oral rehabilitation DMF-T/dmf-t scores of patients who previously had acute rheumatic fevers (*p*=0.33).

Table 4 shows a positive and strong correlation between the pre-oral rehabilitation DMF-T/dmf-t scores and the number of caries patients (r=0.95, *p*=0.01). Additionally, there was no significant relationship between the pre-oral rehabilitation DMF-T/dmf-t scores and the number of missing teeth (r=0.14, *p*=0.09). There was no significant relationship between the pre-oral rehabilitation DMF-T/dmf-t scores and the number of fillings (r=0.11, *p*=0.17). There was no significant relationship between the post-oral rehabilitation DMF-T/dmf-t scores and the number of caries patients. However, a positive and moderately strong correlation was found between the post-oral rehabilitation DMF-T/dmf-t scores and the number of missing teeth (r=0.56, *p*=0.01). In addition, there was a positive and strong relationship between the post-treatment DMF-T/dmf-t scores and the number of fillings (r=0.62, *p*=0.01)

## Discussion

Children with CHD are at high risk of contracting oral diseases. Moreover, the oral diseases of these children are the etiological factors of IE (7). Furthermore, the result of oral-based sepsis could be fatal for these children (8). Therefore, this study investigated these children's oral health background and dental treatment approaches for the prevention of IE. Studies have shown that children with CHD do not present themselves until in the very advanced stage of caries; thus, most dental restorations can be performed under general anaesthesia (GA) due to the advanced dental disease (8,14,19). Therefore, children with CHD, who performed dental rehabilitation under GA, were included in this study. Nevertheless, GA could pose an increased risk to these children's health because it may induce an additional heart condition (6,20). Paediatric cardiologists/surgeons, paediatric dentists, and paediatricians must work together to promote oral health in children at risk for IE so that all practitioners can deliver dental and medical care based on the necessary expertise (8,21). Elghazaway *et al*. determined that identifying inequities and hazards in managing children with cardiac disorders, if addressed effectively by cardiologists and paediatric dentists, may lower the incidence of IE of oral origin (21). Koerdt *et al*. specified that specialized centres with paediatric cardiologists and dentists could coordinate the treatment of oral health conditions and early disease awareness (20). Similarly, Naudi *et al*. stated that children with cardiac issues should be diagnosed in infancy through collaboration with medical colleagues (14). Koerdt *et al*. indicated that an interdisciplinary team containing paediatric cardiologists and paediatric dentists is required to rehabilitate children with CHD (20). In the current study, the authors propose that children with CHD and whose systemic treatment and follow-up was performed by the paediatric cardiology department in the medical university should collaborate with paediatricians for their oral rehabilitation. Heart diseases may be associated with other diseases and syndromes (4). For example, Trisomy 21, 22q11, Noonan syndrome, Turner syndrome, and Williams syndrome account for approximately 21% of CHDs (22). Therefore, the authors decided not to exclude CHD patients with other associated diseases and syndromes, as in a study by Weidner *et al*. (8). In this study, additional diseases were found in 35.2% of the children with CHD. Two studies found caries lesions in groups with cardiac disease (23,24). Tasioula *et al*. found that the mean dmft/DMFT values for children aged 2-16 years were 1.8/0.4 for the healthy group and 1.6/0.8 for those with CHD (25). Stecksen-Blicks *et al*. found that children with CHD, with a mean dmfs value of 5.2 ± 7.0, had significantly more caries than a healthy group, with a mean dmfs value of 2.2 ± 3.5 (*p*<0.05) (3). Pourmoghaddas *et al*. found that the DMFT score was higher in 68 children with CHD compared to 74 healthy children. Nevertheless, their study showed no significant difference (6.4 ± 2.46 vs. 5.5 ± 2.16; *p*=0.14) (26). Karhuma *et al*. found statistically significant differences in both DT (*p*=0.046) and DMFT (*p*=0.009) in children aged 15 years and with different types of cardiac defects (4). Sivertsen *et al*. found that children with CHD and who underwent follow-up were informed about oral rehabilitation experienced less caries and showed lower DMFT scores (11). In this study, the pre-oral rehabilitation DMF-T/dmf-t score of children with CHD was 10.94±4.14, and that after oral rehabilitation was 11.68±4.09 (*p*=0.01). The fact that children with CHD had additional diseases did not significantly change the dmft values. Heart medicines, such as sucrose-based digoxin, ACE-inhibitors, and diuretics, which reduce saliva secretion, promote caries in children with CHD (3,27). In the present study, most children with CHD had used heart medicine. These children showed higher DMF-T/dmf-t scores than those with CHD not using heart medicine during pre-oral rehabilitation (*p*=0.01). Backman *et al*. concluded that heart operations could increase the prevalence of caries in children with CHD (28). Karhuma *et al*. found a trend whereby children who had undergone several heart operations had better caries statuses than others. They determined that the reason for was the number of oral examinations and preventive care (4). In the present study, operated children with CHD showed lower DMF-T/dmf-t scores compared to non-operated children before oral rehabilitation (*p*=0.02). George *et al*. examined the levels of salivary IL-6, a dental caries marker, in children (aged 3-6 years) with VSDs. They found a decrease in IL-6 levels after oral rehabilitation in these children (29). In addition, a study showed that heart diseases, such as ASDs and TOF, were associated with higher DMFT scores (4). In the current study, there were significant differences between the pre- and post-oral rehabilitation DMF-T/dmf-t scores of patients with ASDs, VSDs, mitral regurgitation, bicuspid aortic valves, aortic valve replacements and supravalvular aortic stenosis (with Williams syndrome, myocardial hypertrophy (*p*<0.05). The Paediatric Congenital Heart Disease Standards and Specifications (PCHDSS), which was published in 2016, highlights the prophylaxis IE guidelines. In addition, the PCHDSS emphasises that it is a paediatric dentist cardiology team's responsibility to inform patients of the importance of good oral health. According to this guideline, dentists should inform patients with heart disease of the cardiology of the dental treatments and procedures used to provide oral rehabilitation, to prevent IE (7). For example, a primary tooth with two surfaces affected by caries should be extracted. Likewise, extraction should be considered for primary teeth with severe non-carious tooth surface loss. Similarly, carious teeth should undergo complete caries removal and be definitively restored or extracted (7). It is important to underpin the need for these children to have as little caries and as little pulpal involvement as possible. Therefore, minimally invasive interventions such as the Hall technique should be reconsidered in these children (30). In this study, before oral rehabilitation, the d value was higher at a high baseline DMF-T/dmf-t score in children. After oral rehabilitation, the m and f values were higher at a high DMF-T/dmf-t scores in children. Furthermore, extraction and restorative treatments were used to prevent IE in children with CHD. A limitation of the current study was the absence of a control group for follow-up. Another limitation of the study is that the dentition of the children was not grouped. Paediatric, paediatric cardiology and paediatric dentists should work collaboratively with children with congenital heart disease. Extraction should be considered when providing oral rehabilitation, rather than endodontic and deep restorative treatment.

## Figures and Tables

**Table 1:** Demographic and medical findings of children.

Demographic and medical findings of children			n	%
Demographic Respondents	Gender	Boys	82	56.2
		Girls	64	43.8
	Additional Systemic Diseases	Absent	94	64.4
		Presence	52	35.6
	Heart Diseases	Absent	10	6.8
		Presence	136	93.2
	Using Medicine	Absent	108	74.0
		Presence	38	26.0
	Pre-op oral rehabilitation dmf-t / DMF-T scores	Caries	1557	96.8
		Missing	26	1.7
		Filling	24	1.5
	Post-op oral rehabilitation dmf-t /DMF-T scores	Caries	0	0
		Missing	525	32.7
		Filling	1082	67.3
Comorbidity Diseases	Down Syndrome	-	30	20.5
	Epilepsy	-	13	8.9
	Ehlers Danlos Syndrome	-	6	4.1
	Hypothyroidism	-	6	4.1
	Type 1 diabetes mellitus	-	5	3.4
	Asthma	-	3	2.1
	Attention deficit hyperactivity disorder	-	2	1.4
	Acute lymphocytic leukemia (ALL)	-	1	0.7
	Anemia	-	1	0.7
	Autism	-	1	0.7
Types of Medication Used	Enalapril with Acetyl salicylic acid	-	25	17.1
	Depot penicillin	-	15	10.3
	Propranolol HCl	-	4	2.7
	Furosemide	-	4	2.7
	Metoprolol	-	2	1.4
	Risperidone	-	2	1.4
	Digoxin	-	2	1.4
	Bosentan	-	1	0.7
	Clopidogrel	-	1	0.7
	Atorvastatin	-	1	0.7
	Enoxaparin	-	1	0.7
	Sotalol	-	1	0.7
	Losartan	-	1	0.7
Types of Heart Diseases	Atrial septal defect (ASD)	-	56	38.4
	Ventricular septal defect (VSD)	-	46	31.6
	Supravalvular Aortic Stenosis (with Williams Syndrom)	-	13	8.9
	Bicuspid aorta	-	11	7.5
	Myocardial hypertrophy	-	8	5.5
	Mitral regurgitation	-	4	2.7
	Aortic Valve Replacement	-	2	1.3
	Previous Acute Rheumatic Fever	-	2	1.3
	Arrhythmia	-	2	1.3
	Pulmonary venous return anomaly	-	2	1.3

Note: n: Number, %: Percentage.

**Table 2:** According to children’s properties before/after dmf-t/DMF-T.

Children's properties		dmf-t /DMF-T score before oral rehabilitation		dmf-t /DMF-T score after oral rehabilitation	
Mean±S.D.	p	Mean±S.D.	p
Gender	Boys	10.96±4.22	0.59	11.70±4.20	0.48
	Girls	10.92±4.06		11.66±3.98	
Comorbidity Diseases	Absent	10.98±4.33	0.44	11.52±4.21	0.51
	Presence	10.88±3.80		11.96±3.91	
Using Medicine	Absent	11.27±4.12	0.01*	11.87±4.11	0.39
	Presence	10.03±4.09		11.13±4.05	
Heart Operation	Absent	11.21±4.14	0.02*	11.95±4.01	0.21
	Presence	10.51±4.13		11.24±4.24	

Note: d/D: Decay, m/M: Missing, f/F: Filling, t/T: Teeth, S.D: Standard Deviation, *Significantly p <0.05.

**Table 3:** According to children’s heart diseases before/after dmf-t/DMF-T.

Heart Diseases		dmf-t /DMF-T score before oral rehabilitation		dmf-t /DMF-T score after oral rehabilitation	
Mean±S.D.	p	Mean±S.D.	p
Atrial septal defect (ASD)	Absent	8.01±3.95	0.01*	9.00±3.82	0.01*
	Presence	11.50±3.12		11.45±3.07	
Ventricular septal defect (VSD)	Absent	8.02±3.83	0.01*	8.21+3.71	0.01*
	Presence	11.49±3.15		12.07+3.08	
Mitral regurgitation	Absent	10.98±4.17	0.03*	11.71+4.14	0.03*
	Presence	9.75±2.36		10.5+2.08	
Bicuspid aorta	Absent	11.22±4.11	0.01*	11.93+4.01	0.01*
	Presence	7.55±2.88		8.55+4.01	
Aortic Valve Replacement	Absent	11.00±4.12	0.01*	11.74±4.07	0.01*
	Presence	7.00±4.24		7.00±4.24	
Supravalvular Aortic Stenosis (with Williams Syndrom)	Absent	10.87±4.06	0.03*	11.52±4.00	0.01*
	Presence	11.69±4.94		13.31±4.89	
Previous Acute Rheumatic Fever	Absent	10.95±4.16	0.33	11.68±4.12	0.36
	Presence	10.5±2.12		11.5±0.71	
Myocardial hypertrophy	Absent	12.11±4.11	0.01*	12.83±4.01	0.01*
	Presence	7.17±3.06		8.17±4.79	

Note: d/D: Decay, m/M: Missing, f/F: Filling, t/T: Teeth, S.D: Standard Deviation, *Significantly p <0.05.

**Table 4:** Caries, Missing, Filling scores of dmf-t/DMF-T.

Scores		Caries	Missing	Filling
dmf-t/DMF-t Before Oral Rehabilitation	r	0.95^*^	0.14	0.11
	p	0.01	0.09	0.17
dmf-t/DMF-t After Oral Rehabilitation	r	0.11	0.56^*^	0.62^*^
	p	0.18	0.01	0.01

Note: d/D: Decay, m/M: Missing, f/F: Filling, t/T: Teeth, *Significantly p <0.05.
